# Electrical and Magnetodielectric Properties of Magneto-Active Fabrics for Electromagnetic Shielding and Health Monitoring

**DOI:** 10.3390/ijms21134785

**Published:** 2020-07-06

**Authors:** Madalin Bunoiu, Eugen Mircea Anitas, Gabriel Pascu, Larisa Marina Elisabeth Chirigiu, Ioan Bica

**Affiliations:** 1 Department of Physics, West University of Timisoara, V. Parvan Avenue 4, 300223 Timisoara, Romania; ioan.bica@e-uvt.ro; 2 Joint Institute for Nuclear Research, 141980 Dubna, Russia; anitas@theor.jinr.ru; 3 Horia Hulubei National Institute of Physics and Nuclear Engineering, 077125 Bucharest-Magurele, Romania; 4 Department of Pharmacy, University of Medicine and Pharmacy, 200349 Craiova, Romania; chirigiu_a_larisa@yahoo.com

**Keywords:** iron oxide microfibers, silicone oil, cotton fabrics, electrical properties, magnetic field, electric field

## Abstract

An efficient, low-cost and environmental-friendly method to fabricate magneto-active fabrics (MAFs) based on cotton fibers soaked with silicone oil and iron oxide microfibers (mFe) at mass fractions 2 wt.%, 4 wt.% and 8 wt.% is presented. It is shown that mFe induce good magnetic properties in MAFs, which are subsequently used as dielectric materials for capacitor fabrication. The electrical properties of MAFs are investigated in a static magnetic field with intensities of 0 kA/m, 160 kA/m and 320 kA/m, superimposed on a medium-frequency electric field. The influence of mFe on the electrical capacitance and dielectric loss tangent is determined, and it can be observed that the electrical conductivity, dielectric relaxation times and magnetodielectric effects are sensibly influenced by the applied magnetic and electric fields. The results indicate that the MAFs have electrical properties which could be useful for protection against electromagnetic pollution or for health monitoring.

## 1. Introduction

Recent technological advancements in various fields such as telecommunications, computer science or satellite broadcasts expose human beings to significant increases of the radiation levels of electromagnetic radiations (EMR), especially those arising from the fast-developing 5G technology [1]. Many recent studies have shown that exposure to EMR is causing health hazards for human beings, plants and animals [2,3,4,5] at levels well below most of the accepted international guidelines [6]. The damage goes well beyond living organisms, and includes disruption of the functionality and performance of many electronic devices, slows down their efficiency and degrades the reliability, lifetime and safety of electrical equipment [7,8,9].

Therefore, fabrication of environmental-friendly materials functioning as health monitoring systems and EMR absorbers in a wide bandwidth, produced at low-costs and exhibiting design flexibility, became a stringent necessity nowadays. As a result, many research papers are aimed at investigating smart materials for various specific tasks such as measuring temperature and blood pressure [10], monitoring the pH level of wounds and burns [11], drug delivery [12], electrical stimulation of muscles and posture correction [13], monitoring vital functions for geriatric or disabled patients [14], chronic pain relief [15], movement capture and sleep monitoring [16], cardiac tissue engineering [17], thermal sensing and positioning [18] or thermoelectrics [19]. An important class of smart materials is represented by magneto-active fabrics (MAFs), since they can be designed to include crucial features that enable fabrication of high-performance health monitoring systems and EMR absorbers [20].

Generally, MAFs consist from natural polymeric fibers such as hemp, cotton or bamboo serving as a matrix, soaked with mixtures of artificial polymeric fibers and ferri/ferro-magnetic nano/micro-particles. In particular, polyester fabrics with magnetic properties obtained using a mixture of carbonyl iron (CI) and nano carbon black, along with aluminium sputtering exhibit high microwave absorbing properties, particularly in the primary range of 8.2–12.4 GHz [21]. At a frequency of 14.2 GHz, graphene/Fe nanocomposites with ferromagnetic properties exhibit also significantly enhanced electromagnetic absorption properties as compared with other magnetic materials [22]. These enhanced properties are related to high surface areas of filler/matrix, interfacial polarizations, synergetic effects or an efficient dispersity of the fillers.

In order to further enhance also the magnetic properties, various combinations of the individual components and additives can be used. As such, in Reference [23], a polyester fabric with protective and magnetic properties is fabricated by using a mixture of micro magnetic carbonyl iron powder and nano carbon black through pad-dry-cure method and sputter coating with aluminium (Al). In the same work it has been shown that the presence of nano carbon black and carbonyl iron powder on the polyethylene terephthalate fabric sputter coated with aluminum exhibited higher microwave absorbing properties particularly in the primary range of 8.2–12.4 GHz, as compared with the blank polyethylene terephthalate fabric without Al sputter coating. When using cotton yarns covered with hard (barium hexaferrite) and soft (Black Toner 6745 CP-313) magnetic particles, the magnetic properties (residual magnetism and coercive field intensity) of the yarns increase with the magnetic powder content in the coating solution [24]. However, these improvements are achieved at the expense of some yarn properties, e.g. elasticity. Also, smart textile fabrics obtained through a coating method with NdFeB flake-like microparticles have been shown to have stable magnetic properties, and thus this method can be used to prepare fabrics with high magnetization required under special conditions [25].

For many practical applications required in health monitoring systems and by EMR absorbers, besides good absorption and magnetic properties, the MAFs are required to have also good mechanical, structural and magnetodielectric properties, such as an increased elasticity, complex permittivity/permeability, various structural organizations of the absorbent material, or a matching electromagnetic impedance. Such issues have been partially addressed in Reference [26], where MAFs were synthesized based on cotton cloth soaked with silicone oil (SO), carbonyl iron (CI), and $\gamma -Fe_{2}O_{3}$ particles at various concentrations $\Phi_{\gamma -{\mathrm{Fe}}_{2}O_{3}}$. In the same reference, it has been shown that in the presence of a magnetic field with intensities up to 240 kA/m, superimposed on a medium-frequency electric field, the dielectric properties of the fabrics are sensibly influenced by $\Phi_{\gamma -{\mathrm{Fe}}_{2}O_{3}}$. Furthermore, in Reference [27] it has been shown that for MAFs based on cotton cloth, soaked with SO and CI microparticles at various concentrations $\Phi_{\mathrm{Fe}}$, the relative dielectric permittivity, apparent viscosity, modulus of elasticity, and the components of deformations and of chemical tensions are sensibly influenced by the magnetic field and $\Phi_{\mathrm{Fe}}$.

However, for various bio-medical, technical and industrial applications, an efficient, low-cost and environmental friendly method for fabrication of MAFs is required. To this aim, a first step has been performed recently in Reference [28], where a simple and versatile method to synthesize iron oxide microfibers in large quantities, with high efficiency and good magnetic properties, has been reported. The microfibers consist from iron oxides, and the method is based on the thermal decomposition of iron pentacarbonyl and SO, and vaporization of CI in a microwave plasma.

Here, we use the microfibers obtained by the above microwave-assisted method, and present the fabrication of a high-performance and stable MAFs based on cotton fibers soaked with SO and iron oxide microfibers (mFe) at various mass fractions arranged in a complex fractal structure. Magnetic measurements show good magnetic properties of the obtained MAFs, properties which are induced by mFe. By using MAFs as dielectric materials, a plane capacitor was realized, and it was observed that in a static magnetic field superimposed on a medium-frequency electric field, the electrical conductivity, dielectric relaxation times and magnetodielectric effects are sensibly influenced by the magnetic field intensity and electric field frequency.

## 2. Results

**Figure 1:**
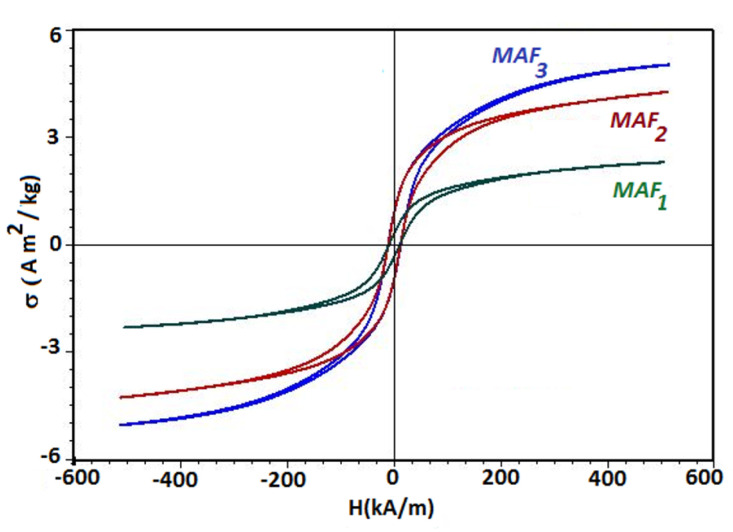
(Color online) Specific magnetization σ of MAFs, as a function of intensity *H* of an external magnetic field, for fixed values of the mass fraction $\Phi_{\mathrm{mFe}}$).

The magnetisation curves of MAFs are shown in Figure 1, and were performed also under sine waveform driving field conditions by means of a laboratory-made ac induction hysteresis graph described in Reference [29]. The saturation specific magnetization occurs at about 477 kA/m, and together with the remanent specific magnetization, they generally increase with the volume fraction of mFe. The numerical values are shown in Table 1. However, the intensity of coercive magnetic field remains unchanged with the mass fraction $\Phi_{\mathrm{mFe}}$, and its value is about 38 kA/m. Note that the obtained MAFs consist from a mixture of different components, i.e., cotton fibers and silicone oil, in addition to the microfibers, and this gives rise to an apparent specific magnetization in Figure 1 and Table 1. On average, they are one order of magnitude higher than that of α-Fe${}_{2}$O${}_{3}$ (hematite), but one (or even more) order(s) of magnitude smaller as compared to γ-Fe${}_{2}$O${}_{3}$ (maghemite) and Fe${}_{3}$O${}_{4}$ (magnetite) [30]. This indicates that the microfibers forming the MAFs, consist of phases with much larger saturation magnetization as compared to hematite.

### 2.1. Fabrication of the Electrical Device Based on MAFs

**Figure 2:**
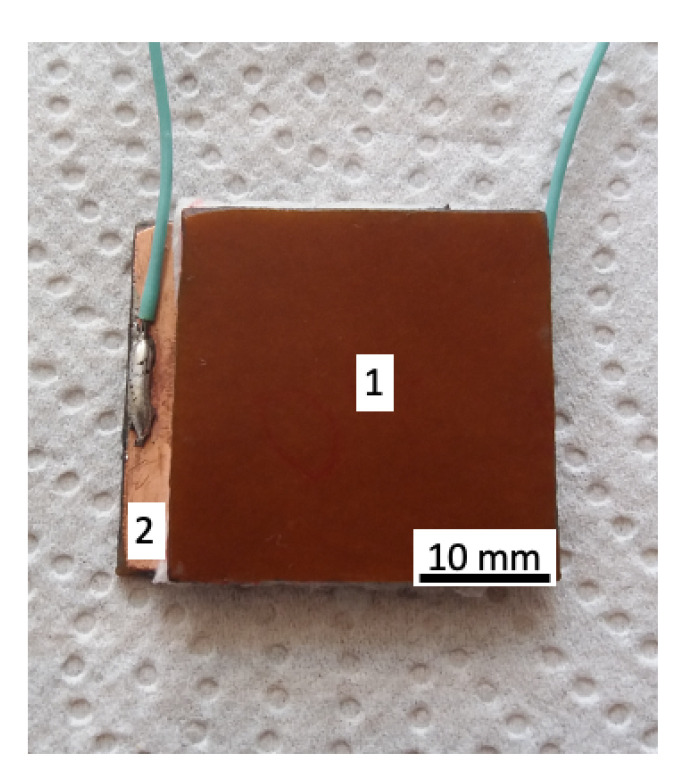
(Color online) Electrical device (ED) consisting of two parallel textolite plates (1) arranged such that the MAF is situated between their copper-side (2).

The electrical device (ED) used to investigate the electrical and magnetodielectric properties of MAFs in a magnetic field is shown in Figure 2. It consists from two parallel textolite plates in the shape of a square with the edge length of 30 mm (pos. 1). Between the copper-sides of the two textolite plates (pos. 2) is placed the MAF. The obtained system is referred thereafter as ED.

### 2.2. Experimental Setup and Measurements

**Figure 3:**
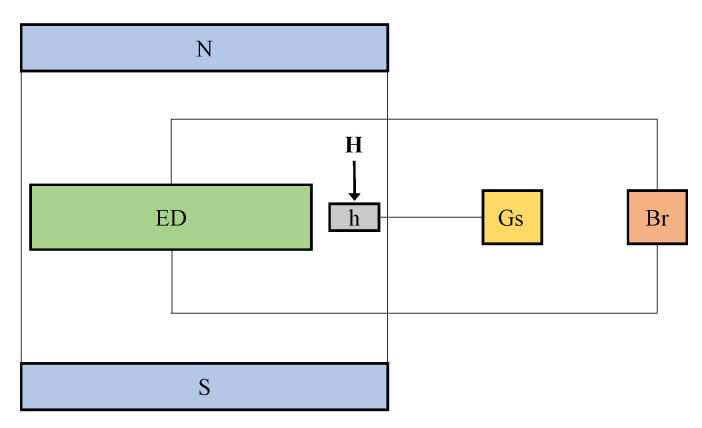
(Color online) Experimental setup: N and S—poles of the electromagnet, ED—electrical device, Br—RLC-bridge, Gs—gaussmeter, h—Hall probe.

The configuration of the experimental setup used is shown in Figure 3. The setup consists from a continuous source electromagnet (not shown here), powered by a continuous current source, RXN-3020Dt type, from Electronics Co. LTD. The ED is fixed between the N and S poles of the electromagnet, which is electrically connected to the bridge Br, E7-20 type from MNIPI. A Hall probe of the gaussmeter, DX-102 type from Dexing Magnet, is fixed on the ED.

Inside the ED are introduced by turn, the double-layered cotton fabrics, a system consisting from cotton fabrics and 0.8 cm${}^{3}$ SO, and respectively MAFs. The ED is fixed between the poles of the electromagnet, and its thickness is fixed at 1 mm. By this, one creates a good electrical contact between the samples and the copper electrodes of ED. The intensity of the electric current through the coil of the electromagnet is fixed such that the deviation of the magnetic field intensity *H* is kept within ±5% from the selected value. The RLC bridge Br is configured such that it measures the equivalent electrical components connected in parallel. The equivalent electrical capacitance $C_{p}$ of the ED is measured with a precision of ±0.1 %, while the dielectric loss tangent $D_{p}$ is measured with a precision of ±0.001%. The electrical impedance at the terminals of the RLC bridge is 10 kΩ. The electrical voltage applied to the ED is fixed at 1 V${}_{\mathrm{ef}}$.

### 2.3. Equivalent Capacitance and Dielectric Loss Tangent

**Figure 4:**
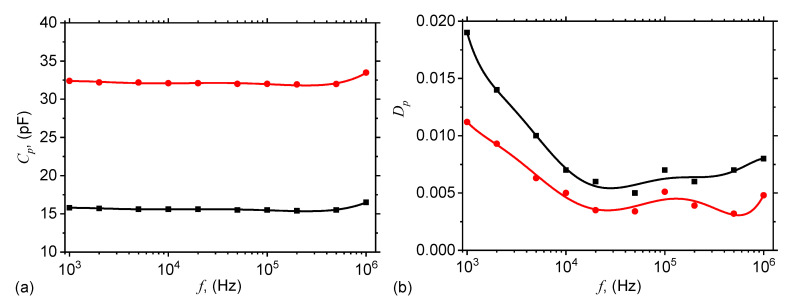
(Color online) Equivalent electrical capacitance $C_{p}$ (**a**), and the dielectric loss tangent $D_{p}$ (**b**) of the ED with cotton fabrics (black), and of the ED with cotton fabrics + SO (red) as a function of the frequency *f* of the alternating electric field. Dots—experimental data; Continuous curves—polynomial fits.

The equivalent electrical capacitance $C_{p}$ and the dielectric loss tangent $D_{p}$ of the ED are measured at frequencies 1 kHz, 2 kHz, 5 kHz, 20 kHz, 50 kHz, 100 kHz, 200 kHz, 500 kHz and 1000 kHz. For cotton fabrics, and cotton fabrics + SO, there is no external magnetic field applied, and the results are presented in Figure 4.

**Figure 5:**
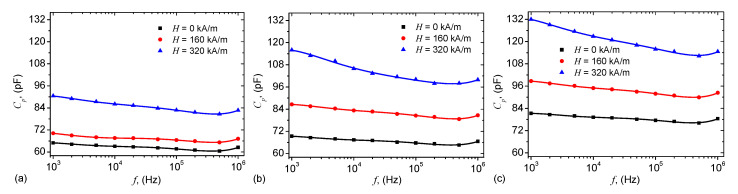
(Color online) Equivalent electrical capacitance $C_{p}$ as a function of the frequency *f* of the alternating electric field. (**a**) MAF${}_{1}$; (**b**) MAF${}_{2}$; (**c**) MAF${}_{3}$. Dots—experimental data; Continuous curves—polynomial fits.

**Figure 6:**
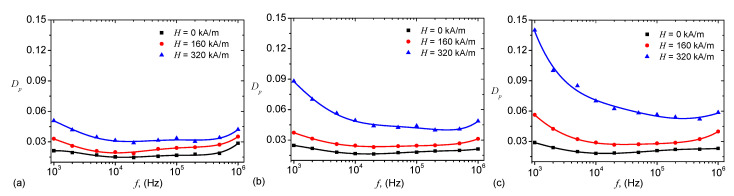
(Color online) Dielectric loss tangent $D_{p}$ as a function of the frequency *f* of the alternating electric field. (**a**) MAF${}_{1}$; (**b**) MAF${}_{2}$; (**c**) MAF${}_{3}$. Dots—experimental data; Continuous curves—polynomial fits.

For the MAF${}_{i}$, i=1,2,3, the variation of $C_{p}$ and $D_{p}$ with frequency *f*, and at fixed values of intensity *H*, is shown in Figure 5, and respectively in Figure 6.

**Figure 7:**
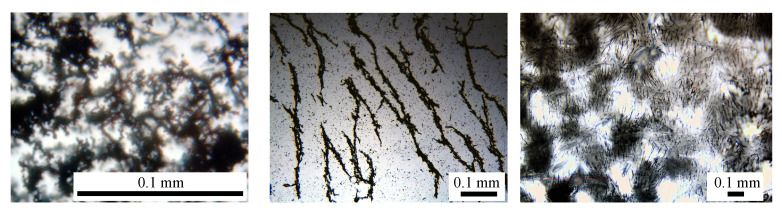
(Color online) (**a**) Microfibers mFe (dark regions) mixed with silicone oil SO (light regions), without an applied magnetic field; (**b**) mFe (dark regions) mixed with SO (light regions), with an applied magnetic field. The dark spots are individual fibers which were not attached to the chains. (**c**) Cotton fibers cotton fabrics dark regions with SO (light regions) and mFe (light blue chain-like regions) in a magnetic field which points upwards through paper/screen.

The structural changes and formation of magnetic dipoles in the presence of an external magnetic field, responsible for the observed effects for the capacitance $C_{p}$ in Figure 4, and for dielectric loss tangent $D_{p}$ in Figure 5, are presented in Figure 7, which shows the mixture of microfibers mFe (dark regions) and SO (light regions) without the presence of an external magnetic field.

### 2.4. Influence of the Microfibers to Electrical Capacitance and Dielectric Loss Factor

**Figure 8:**
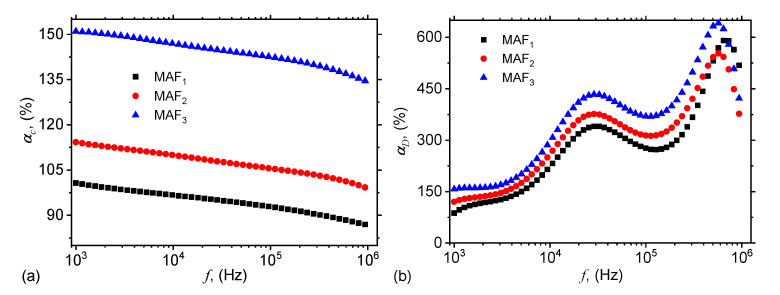
(Color online) Variation of $\alpha_{C}$ (**a**) and $\alpha_{D}$ (**b**) of MAFs, with the frequency *f* of the alternating electric field.

The influence of the microfibers to variation of the equivalent electrical capacitance with the frequency can be expressed qualitatively, through the quantity:

(1) $$\alpha_{C}\left(\%\right)=\frac{C_{p}\left(f,H\;=0\right)}{C_{p}^{cotton+SO}\left(f\right)}\times 100,$$

where $C_{p}\left(f,H\;=0\right)$ is the capacitance as a function of frequency at H=0 from Figure 5, and $C_{p}^{cotton+SO}\left(f\right)$ is the capacitance of the ED with cotton fabrics and SO from Figure 4a. Therefore, the variation of $\alpha_{C}$ with the frequency *f* has the behavior shown in Figure 8a.

The contribution of the microfibers to $D_{p}$ can be quantified in a similar manner, that is:

(2) $$\alpha_{D}\left(\%\right)=\frac{D_{p}\left(f,H\;=0\right)}{D_{p}^{cotton+SO}\left(f\right)}\times 100,$$

where $D_{p}\left(f,H\;=0\right)$ is the dielectric loss tangent as a function of frequency at H=0 from Figure 6, and $D_{p}^{cotton+SO}\left(f\right)$ is the dielectric loss tangent of ED with cotton fabrics and SO from Figure 4b. Therefore, the variation of $\alpha_{D}$ with the frequency *f* has the behavior shown in Figure 8b.

### 2.5. Electrical Resistance

The results obtained in Figure 4, Figure 5 and Figure 6 suggest that the electrical device ED can be assimilated to an electrical circuit (see also Reference [26]). At the terminals of the circuit, a variable voltage of the form $u=U\exp\left(j2\pi ft\right)$ is applied, where: *U* is the amplitude, $j=\sqrt{-1}$, *f* is the frequency, and *t* is the time. Thus, the current through the circuit has the intensity:

(3) $$I^{*}=j2\pi f\epsilon_{r}^{*}C_{p0}u,$$

where $\epsilon_{r}^{*}=\epsilon_{r}^{{}^{\prime }}-j\epsilon_{r}^{{}^{\prime \prime }}$, $\epsilon_{r}^{{}^{\prime }}$ is the relative dielectric constant, $\epsilon_{r}^{{}^{\prime \prime }}$ is the dielectric loss factor, and $C_{p0}$ is the equivalent electrical capacitance of the ED for $\epsilon_{r}^{{}^{\prime }}=1$.

In the case of a plane capacitor, the latter quantity is given by:

(4) $$C_{p0}=\frac{\epsilon_{0}S}{d},$$

where $\epsilon_{0}$ is the vacuum dielectric constant, *S* is the area of the common surface of copper electrodes, and *d* is the distance between them. By introducing in Equation (3), the capacitance given by Equation (4), one obtains:

(5) $$I^{*}=2\pi f\epsilon_{r}^{{}^{\prime \prime }}\frac{\epsilon_{0}S}{d}u+j2\pi f\epsilon_{r}^{{}^{\prime }}\frac{\epsilon_{0}S}{d}u.$$

The admittance of the circuit is given by $Y\equiv I^{*}/u=1/R_{p}+j2\pi fC_{p}$. By using the expression given by Equation (5), one obtains:

(6) $$1/R_{p}+j2\pi fC_{p}=2\pi f\epsilon_{r}^{{}^{\prime \prime }}\frac{\epsilon_{0}S}{d}u+j2\pi f\epsilon_{r}^{{}^{\prime }}\frac{\epsilon_{0}S}{d}u.$$

Thus, by identifying the real and imaginary parts in the above relation, one obtains the components of the relative dielectric permittivity as:

(7) $$\epsilon_{r}^{{}^{\prime }}=\frac{C_{pd}}{\epsilon_{0}S},$$

and respectively:

(8) $$\epsilon_{r}^{{}^{\prime \prime }}=\frac{d}{2\pi f\epsilon_{0}SR_{p}}.$$

It is well known that the dielectric loss tangent is related to the components of the dielectric permittivity by [31]:

(9) $$\epsilon_{r}^{{}^{\prime \prime }}=D\epsilon_{r}^{{}^{\prime }}.$$

Therefore, by using Equations (7) and (8), together with Equation (9), the equivalent electrical resistance $R_{p}$ can be written as:

(10) $$R_{p}=\frac{1}{2\pi fC_{p}D}.$$

**Figure 9:**
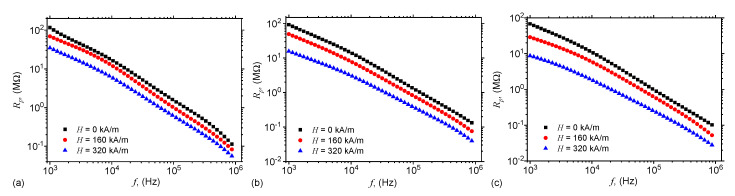
(Color online) Equivalent electrical resistance $R_{p}$ as a function of the frequency *f* of the alternating electric field. (**a**) MAF${}_{1}$; (**b**) MAF${}_{2}$; (**c**) MAF${}_{3}$.

By introducing in this relation the variation of the capacitance $C_{p}$ from Figure 4, and the dielectric loss tangent $D_{p}$ from Figure 6, one finally obtains the variation of $R_{p}$ with the frequency *f*, as presented in Figure 9.

### 2.6. Dielectric Relaxation Times

Figure 5 and Figure 9 show that for each frequency *f* of the electric field, there exists a pair of values $\left(C_{p},R_{p}\right)$ for the equivalent electrical scheme. Thus, one can associate a time constant τ to this circuit, which can be approximated by the dielectric relaxation time, i.e.,

(11) $$\tau =C_{p}R_{p}.$$

**Figure 10:**
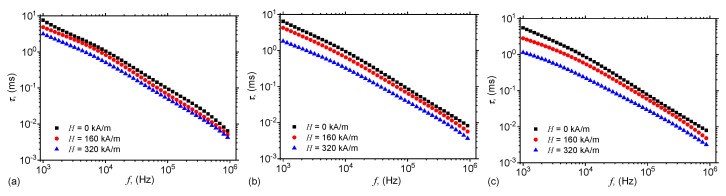
(Color online) Dielectric relaxation times τ as a function of the frequency *f* of the alternating electric field. (**a**) MAF${}_{1}$; (**b**) MAF${}_{2}$; (**c**) MAF${}_{3}$.

By using the functions $C_{p}$ and $R_{p}$ from Figure 5 and Figure 9, one obtains in Figure 10 the variation of dielectric relaxation time with the frequency *f*, for fixed values of magnetic field intensity *H* and mass fractions $\Phi_{\mathrm{mFe}}$.

### 2.7. Magnetodielectric Effects

**Figure 11:**
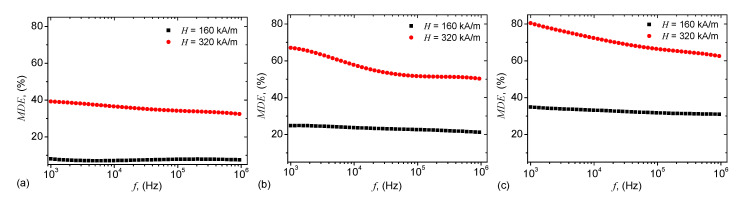
(Color online) Magnetodielectric effects $MDE\;\left(\%\right)$ as a function of the frequency *f* of the alternating electric field. (**a**) MAF${}_{1}$; (**b**) MAF${}_{2}$; (**c**) MAF${}_{3}$.

The magnetodielectric effect is defined by:

(12) $$MDE\left(\%\right)=\left(\frac{C_{p}{\left(f,H\;\right)}_{\Phi_{\mathrm{mFe}}}}{C_{p}{\left(f,H=0\right)}_{\Phi_{\mathrm{mFe}}}-1}\right)\times 100$$

where $C_{p}{\left(f,H\;\right)}_{\Phi_{\mathrm{mFe}}}$ and $C_{p}{\left(f,H=0\right)}_{\Phi_{\mathrm{mFe}}}$ are the equivalent capacitances of MAFs with, and respectively without a magnetic field. By using the capacitances from Figure 4a and Figure 5 in Equation (12), one obtains the magnetodielectric effects shown in Figure 11.

## 3. Discussion

The results in Figure 4a show that $C_{p}$ of the ED with cotton fabrics (black), and respectively of the ED with cotton fabrics + SO (red) are quasi-constants with frequency *f* of the electric field. However, for cotton fabrics + SO the $C_{p}$ is about twice higher, since this system can be assimilated to two plane capacitors, one having cotton fabrics as dielectric material, and the other one having SO as dielectric material. In this case, the end effect is the sum of the two individual effects, and this results is an increase of $C_{p}$, as shown in Figure 4a.

The dielectric loss tangents $D_{p}$ of the ED with cotton fabrics (black), and respectively of the ED with cotton fabrics + SO (red) are presented in Figure 4b and in both cases, one can observe a decrease up to $f\approx 2\times 10^{4}$ Hz, followed by a region with oscillating values, up to $f=10^{6}$ Hz. However, in the case of the ED with cotton fabrics, $D_{p}$ is up to two times higher on the whole measured range. This is due to the fact that cotton fabrics have fibers with their own intrinsic humidity, to which is added the humidity from the environment. The smaller values of the ED with cotton fabrics + SO are also due to the presence of SO, as shown also in Reference [32].

By comparing the results in Figure 5 with those from Figure 4a one can see that the variation of the capacitance $C_{p}$ with frequency *f* is sensibly influenced by the mass fraction $\Phi_{\mathrm{mFe}}$ of microfibers, and also by the magnetic field intensity *H*. In all cases, the variation is quasi-linear up to f≈500 kHz, where an inflection point arises. In the presence of the magnetic field, the fibers are aligned along the magnetic field lines, as shown in Figure 7. Formation of such aggregates inside the liquid/viscoelastic matrices or fibers lead to electrical properties which are sensibly changed by the magnetizable phase even at low concentrations, and are also sensibly influenced by an external magnetic field intensity [26,27,31,33,34,35,36,37,38,39,40].

Figure 5 shows that, for a fixed frequency *f*, the dielectric loss factor $D_{p}$ increases with intensity magnetic field intensity *H*, in a similar manner as the capacitance $C_{p}$ from Figure 5. This behaviour is attributed to formation of magnetic dipoles aggregates inside cotton fabrics + SO, and has been observed also in other systems, as described in References [34,37,38,41]. The numerical values of the capacitance obtained in Figure 5 are comparable, at the same volume of liquid solutions $S_{i}$ and the same common surface area of the capacitor plates, with those obtained in References [26,27]. However, here the quantity of magnetizable phase, i.e., iron oxide microfibers, is much lower.

However, at a fixed value of *H*, variation of $D_{p}$ with *f* shows a succession of maxima and minima which are dampened with increasing the quantity of microfibers in MAFs. Note that although the mass fraction of the microfibers is small, the increase of $D_{p}$ is enough pronounced such that the absorbing properties of MAFs are sensibly changed. This feature makes the obtained MAFs good candidates for electromagnetic shielding in the investigated range of frequencies.

It is clear from Figure 7a that without a magnetic field, the microfibers form complex aggregates without any preferential order. However, when a magnetic field of intensity H is applied, the microfibers tend to align in the direction of the magnetic field lines, as shown in Figure 7b. Finally, when the mixture of microfibers and SO is embedded in the cotton fibers, and in the presence of a magnetic field, the microfibers also arrange themselves along the magnetic field lines. However, they are stuck to the cotton fibers yarns, and thus formation of aggregates is avoided (see also Reference [27]).

The common feature of $\alpha_{D}$ and $\alpha_{C}$ is that they both increase with the mass fraction of microfibers. However, the type of variation is different: while the functions $\alpha_{C}$ have a quasilinear decrease with the frequency *f*, the functions $\alpha_{D}$ increase with *f* through a succession of maxima and minima. The heights of these maxima are related to the quantities of microfibers inside MAFs, and the observed effects arise due to the increase of the density of electroconductive magnetic dipoles along the magnetic field lines. Similar behavior has been observed also in membranes based on cotton fibers with carbonyl iron microparticles and SO [26,27], or honey [34].

For magnetic materials consisting from ferri/ferro-magnetic nano/micro-particles dispersed in liquid/elastic matrices, as the MAFs obtained here, the equivalent electrical resistance $R_{p}$ and capacitance $C_{p}$ arise due to the interaction between magnetic dipoles from the chains and due to the concentration of the magnetizable phase [33,35,36,39,40]. Along the magnetic dipoles chains, oriented in the direction of H, and chosen in such a way that it coincides with axis Ox, the equivalent electrical capacitance $C_{x}$ between two neighbouring dipoles, and the equivalent resistance $R_{x}$ between dipoles are given by [26] $C_{p}=\pi \epsilon_{0}\epsilon_{r}^{{}^{\prime }}d^{2}/\left(4x\right)$, and respectively $R_{x}=4x/\left(\pi d^{2}E_{C}\right)$, where *x* is the distance between center-of-masses of dipoles, $E_{C}$ is the electrical conductivity, and *d* is the average diameter of the magnetic dipoles, here the thickness of the dipoles consisting from mFe.

Also, it is known that the distance between the center-of-masses of dipoles can be approximated by [26]: $x≃{\left(\delta^{5}-1.25\mu_{0}d^{5}H^{2}t\eta^{-1}\right)}^{1/5}$, where $\delta ≃d\Phi_{\mathrm{mFe}}^{-1/3}$ is the initial distance between microfibers, *t* is the time duration for which the magnetic field is applied, and η is the viscosity of cotton fabrics with SO. Indeed, by increasing $\Phi_{\mathrm{mFe}}$, δ decreases, and for fixed values of *H* and η, the distance *x* decreases, which leads to an increase of $C_{x}$ and a decrease of $R_{x}$. Indeed, Figure 4 and Figure 9 reflect this behavior. However, for a fixed value of the quantity of microfibers, the initial distance between the magnetic dipoles remains constant, and this leads to an increase of the equivalent capacitance (Figure 5), and respectively to a decrease of the equivalent resistance (Figure 9). Note that the effects shown in Figure 4 and Figure 9 are obtained by using much lower quantities of microfibers, as compared with the quantities of carbonyl iron microparticles used to obtain similar effects in References [26,27,33,35,36,39,40,41].

Figure 10 shows that for a fixed value of the magnetic field intensity *H*, the dielectric relaxation times decrease with increasing the mass fraction $\Phi_{\mathrm{mFe}}$. However, for fixed values of $\Phi_{\mathrm{mFe}}$, the relaxation dielectric times decrease with increasing the intensity *H*, since the intensity of magnetic interaction between the magnetic dipoles inside a chain is related to the magnetic field intensity *H* by [31] $F_{\mathrm{mag}}\propto H^{2}$. By increasing *H*, the chains consisting from microfibers become more rigid, and the mixture consisting from cotton fabrics and SO becomes more viscous. In the presence of a static magnetic field superimposed on an alternating electric field with $\omega >10^{5}$ s${}^{-1}$, the electric dipoles are only slightly influenced by the variation of the electric current. This leads to an increase of the dielectric loss, and the polarization mechanism changes from a relaxation to an interfacial one. As a consequence, the electrical conductivity of MAFs increases, as shown in Figure 9.

Figure 11 shows that, for a fixed value of magnetic field intensity *H*, MDE increases significantly with increasing the mass fraction $\Phi_{\mathrm{mFe}}$ of microfibers. However, for a fixed value of $\Phi_{\mathrm{mFe}}$, the MDE increases with increasing magnetic field intensity. This increase arise due to the magnetic interactions between magnetic dipoles, and it is influenced by modification of the viscosity of the mixture of SO with microfibers. A similar effect has been observed also in mixtures of SO with carbonyl iron microparticles (see also References [26,27]). By applying an electric field of medium frequency over a static magnetic field, one can observe that the MDE varies with the frequency *f* as shown in Figure 11.

## 4. Materials and Methods

### 4.1. Fabrication of mFe

The mFe are synthesized by following the procedure described in Reference [28]. This involves introducing a mixture of carbonyl iron, C-3518 type, and iron pentacarbonyl, both from Sigma-Aldrich (Germany), mixed with SO, MS100 type, from Silicone Commerciale SpA (Italy) with kinematic viscosity $v_{\mathrm{CI}}=100$ cSt and mass density $\rho_{SO}=0.97$ g/cm${}^{3}$ at 298 K, in a microwave field (2.45 GHz/ 450 W) for about 120 s.

Scanning electron microscopy (SEM) show a complex spiderweb-like structural organization of microfibers, of a multifractal type. The microfiber have a nonuniform surface and their diameters are between 0.25 and 2.20 μm [28]. Magnetic measurements show a saturation specific magnetization of 22.7 Am${}^{2}$/kg, and is obtained when the magnetic field intensity is 477 kA/m. The remanent specific magnetization is 2.86 Am${}^{2}$/kg, and the intensity of coercive magnetic field is 16.67 kA/m. EDX spectrum for elemental analysis of the microfibers indicates that they consist of iron oxides [28].

### 4.2. Fabrication of MAFs

**Figure 12:**
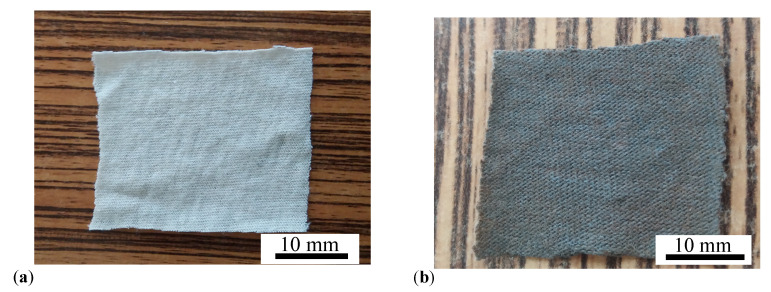
(Color online) (**a**) Double layered cotton fabrics, with dimensions 30mm×30mm×1.20mm. (**b**) MAF.

The materials used for fabrication of MAFs are SO and mFe described above, together with cotton fabrics with a mass density of 0.306 g/cm${}^{3}$, from MedAz (Romania). The cotton fiber is a moderately strong fiber and its tensile strength is similar to common fibers used for fabrication of textile materials. The main steps in preparing the MAFs are the following:

1. A quantity of 4.6 g of SO and 0.4 g of mFe are mixed in a Berzelius glass beaker placed on a heater. When the temperature reaches about 423 K, the homogenisation of the mixture is continued for about 300 s. As such, the humidity present in mFe is eliminated. At the end of this step the mass fraction of mFe is $\Phi_{\mathrm{mFe}}=8$ wt. %. The obtained sample is denoted by S${}_{3}$;
2. A quantity of 2.5 g of SO is poured into a Berzelius glass beaker, to which is added 2.5 g of $S_{3}$ containing 0.2 g mFe and 2.3 g of SO. Thus, one obtains a liquid sample containing 4.8 g of SO and 0.2 g of mFe, and which is denoted $S_{2}$. Thus, the mass fraction of mFe is $\Phi_{\mathrm{mFe}}=4$ wt. %;
3. A quantity of 2.5 g of SO is poured into a Berzelius glass beaker, to which is added 2.5 g of $S_{2}$ containing 0.1 g mFe and 2.4 g of SO. Thus, one obtains a liquid sample containing 4.9 g of SO and 0.1 g of mFe, and which is denoted $S_{1}$. Thus, the mass fraction of mFe is $\Phi_{\mathrm{mFe}}=2$ wt. %. Table 2 summarizes the composition of samples $S_{1},S_{2}$ and $S_{3}$;
4. From the cotton fabric, with a height of 0.6 mm, six pieces are cut in the form of a square with edge length of 30 mm, as shown in Figure 12a. Out of the six pieces, we form three double-layered structures, by superimposing two pieces on top of each other;
5. On top of each double-layered structure is deposited a volume of 0.8 cm${}^{3}$ of liquid solutions $S_{i}$, with compositions given in Table 2. Thus, one obtains three MAFs consisting of cotton fibers soaked with SO and mFe, as shown in Figure 12b, denoted MAF${}_{1}$, when $\Phi_{\mathrm{mFe}}=2$ wt. %, denoted MAF${}_{2}$, when $\Phi_{\mathrm{mFe}}=4$ wt. %, and respectively MAF${}_{3}$, when $\Phi_{\mathrm{mFe}}=8$ wt. %.

Since the mechanical properties of cotton fiber are not affected by addition of mFe and SO, the overall mechanical properties of MAFs are at least comparable with those of cotton fabrics. Similar effects have been observed in polyester fabrics with durable photo-, bio-, and magneto-activities, and where the tensile properties of the treated samples were enhanced as compared to the untreated fabric [42].

## 5. Conclusions

In this paper we present the synthesis of magneto-active fabrics based on cotton fibers in the form of gauze bandage, soaked with silicone oil and iron oxide microfibers at various mass concentrations $\Phi_{\mathrm{mFe}}$. The fabrics are used as dielectric materials for fabrication of plane capacitors.

We show that in a static magnetic field of intensity *H* superimposed on an alternating electric field of frequency *f*, the equivalent electrical capacitance and the equivalent electrical resistance of the capacitor are sensibly influenced by H,f and $\Phi_{\mathrm{mFe}}$. In particular, it is shown that for a fixed value of *f*, an increase of $\Phi_{\mathrm{mFe}}$ and *H* leads to a decrease of dielectric times (Figure 10), and to a significant increase of relative dielectric permittivity (Figure 5), dielectric loss tangent (Figure 6) and of the magnetodielectric effects (Figure 11). The latter ones are characterized by a succession of maxima and minima, more pronounced at smaller values of *H* and $\Phi_{\mathrm{mFe}}$, and whose positions are determined by the frequency *f*.

The magnetodielectric effects exhibited by the obtained magneto-active tissues, together with the good magnetic and mechanical properties induced by the iron oxide microfibers, and respectively by the cotton fibers, make them very promising candidates in the fabrication of health monitoring systems and EMR absorbers.

## Figures and Tables

**Table 1:** The apparent saturation specific magnetization ($\sigma_{s}$), and the apparent remanent specific magnetisation ($\sigma_{r}$) for the MAFs at different values of mass fractions $\Phi_{m\mathrm{Fe}}$, extracted from Figure 1.

Sample	$\sigma_{s}$ (Am${}^{2}$/kg)	$\sigma_{r}$ (Am${}^{2}$/kg)	$\Phi_{m\mathrm{Fe}}$ (wt. %)
$MAF_{1}$	2.21	0.4	2.0
$MAF_{2}$	4.37	0.95	4.0
$MAF_{3}$	6.51	0.95	8.0

**Table 2:** The mass $m_{\mathrm{SO}}$ of SO, $m_{\mathrm{mFe}}$ of mFe, and mass fraction $\Phi_{\mathrm{mFe}}$ of mFe inside the liquid solutions $S_{i}$, i=1,2,3.

Sample	$m_{\mathrm{SO}}$ (g)	$m_{\mathrm{mFe}}$ (g)	$\Phi_{\mathrm{mFe}}$ (wt. %)
$S_{1}$	4.9	0.1	2.0
$S_{2}$	4.8	0.2	4.0
$S_{3}$	4.6	0.4	8.0

## Abbreviations

The following abbreviations are used in this manuscript:

MAF	Magneto-active fabric
mFe	microfibers
SEM	scanning electron microscopy
EDX	energy-dispersive X-ray spectroscopy
XRD	X-ray diffraction
EMR	electromagnetic radiation
SO	silicone oil
ED	electrical device
MDE	magnetodielectric effect
